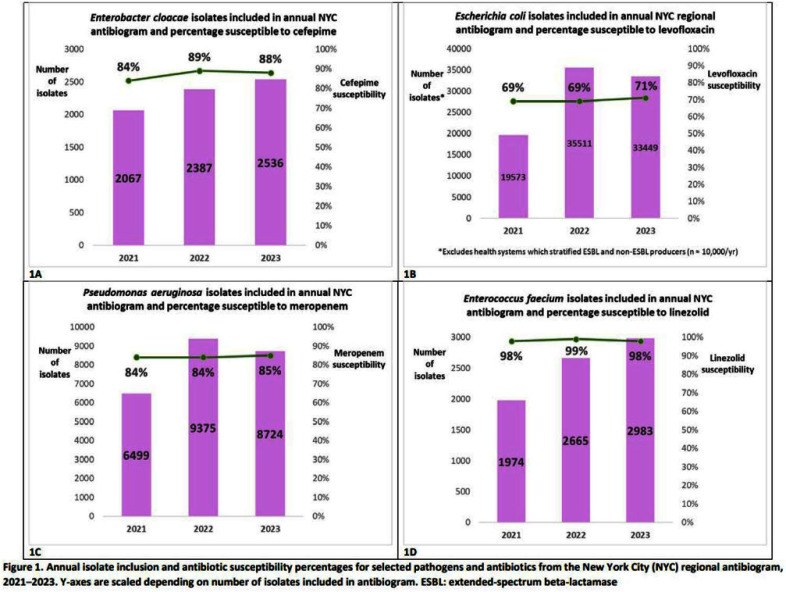# Creation and Distribution of 2021–2023 New York City Regional Antibiograms

**DOI:** 10.1017/ash.2025.381

**Published:** 2025-09-24

**Authors:** William Greendyke, Elise Mantell, Emma Moore, Elizabeth Cave, Molly Kratz

**Affiliations:** 1 New York City Health Dept; 2 New York City Department of Health and Mental Hygiene; 3 NYC DOHMH; 4 NYC Department of Health & Mental Hygiene

## Abstract

**Background:** New York City (NYC) is vulnerable to antimicrobial resistant pathogens given its population density, mobile population of travelers and immigrants, and multiple health systems in close proximity. While some NYC health systems make institutional antibiograms publicly available, it remains challenging for clinicians, antimicrobial stewardship programs, and public health institutions to understand regional antimicrobial resistance trends. Multifacility antibiograms can raise awareness of regional trends in resistance and potentially serve as a benchmark for local facilities. We created and distributed an annual regional antibiogram based on facility-level data from NYC healthcare systems. **Method:** Using Clinical Laboratory Standards Institute guidance, facility antibiograms for calendar years 2021, 2022, and 2023 were solicited by the NYC Health Department and voluntarily submitted by NYC healthcare systems. NYC regional antibiograms were generated for each calendar year by Firstline (Firstline.org, Vancouver, BC), a vendor with technical expertise in creating multifacility antibiograms. When sufficient data were received and facility confidentiality could be ensured, data were stratified to create additional antibiograms by facility borough, setting (i.e., emergency departments), or patient type (i.e., pediatric patients). Antibiogram data were uploaded to Firstline’s clinical decision support application for patient-facing NYC prescribers. **Result:** Of 56 NYC hospitals, 45 (80%) submitted antibiogram data during the project period, comprising 19,766/25,929 (76%) of NYC hospital beds. Of these hospitals, 40 (89%) submitted antibiogram data suitable for inclusion in >1 year of the citywide antibiogram (average: 31 hospitals/year). Annual antibiograms were created for Manhattan, Brooklyn, and Queens; insufficient data were received to create borough-level antibiograms for Staten Island (except 2022) and the Bronx. Annual pediatric and emergency department antibiograms were generated from citywide data. Citywide resistance rates for select pathogens and antibiotics appeared stable across the 3 years (Figure 1A-D). Antibiogram data received 417 views on Firstline between November 14, 2023–December 18, 2024. **Conclusion:** Through voluntary antibiogram submission from health systems, the NYC Health Department generated annual citywide antibiograms that comprised the majority of hospital beds in NYC. We achieved a high rate of voluntary participation because health systems submitted existing institutional antibiograms. Additionally, distributing the NYC antibiogram via a clinical decision support application allowed clinicians to access up-to-date citywide antimicrobial resistance rates in NYC. Despite high participation, differences in data reporting limited our ability to pool antibiograms across facilities (e.g., Figure 1B), reducing representativeness. In future antibiogram iterations, in addition to increasing facility participation, we will explore using susceptibility data electronically reported to the Health Department.

**Figure:**